# The experiences of autistic medical students in relation to seeking and receiving online support: A phenomenological study

**DOI:** 10.1371/journal.pone.0345156

**Published:** 2026-03-20

**Authors:** Samantha Cooper, Mary Doherty, Sebastian Charles Keith Shaw

**Affiliations:** 1 Department of Medical Education, Brighton and Sussex Medical School, Brighton, East Sussex, United Kingdom; 2 UCD School of Medicine, University College Dublin, Dublin, Ireland; University of Warsaw, POLAND

## Abstract

**Background:**

Previous studies have reported that autistic medical students experienced specific challenges throughout their medical studies, including isolation, bullying, and discrimination from their institutions – alongside receiving minimal support. Despite this, it has been argued that many autistic characteristics are well aligned with practising medicine, bringing key benefits to patient care. **‘**Autistic Medical Students’ (AMS) is an online international support group, containing over 200 members – a sub-group of Autistic Doctors International. This study aims to explore the experiences of AMS membership and if membership has any wider impacts on medical studies or wellbeing. Within this, it aims to explore the experiences that led to seeking this support.

**Methods:**

This was a qualitative study. Five participants from AMS were recruited. Semi-structured, 1:1 interviews were conducted via Zoom. These were audio recorded, transcribed verbatim, and analysed using interpretive phenomenological analysis.

**Results:**

Group experiential themes included: medical culture, belonging, safety, ability to thrive, internalised optimism for the future, and real modelling. Participants experienced systemic ableism and weaponised professionalism, which led to a sense of helplessness for their futures. AMS had a positive impact on participants. They experienced a sense of belonging, alongside an improved ability to both recognise their own needs and implement accommodations. They also felt better able to advocate for support. ‘Real modelling’ from peers and autistic doctors motivated participants in their wider medical studies. Of particular benefit was that AMS uniquely encompassed both being autistic and a medical student, making its specific support invaluable.

**Discussion:**

Findings were in keeping with previous research. Autistic peer-to-peer communication enabled development of social relationships and self-identity. Autistic medical students and doctors exhibit traits that make them assets to medicine. This study highlights the need for knowledge, acceptance and representation of autism within medical education – to enable autistic medical students to thrive.

## Introduction

The neurodiversity paradigm considers that autism – alongside other neurodevelopmental differences – are not ‘disorders’ but instead a natural diversity of human cognition [1]. This paradigm rejects the concept that neurotypical people – those with neurocognitive functioning that is considered ‘normal’ by society – are the standard that all people should be measured against [2]. As such, this reframes ‘deficits’ as differences, where autistic characteristics may indeed still manifest as challenges, but recognising that this relates to a discrepancy between intrinsic autistic differences and external societal factors [3]. This also allows for the acknowledgement that autistic difference can also bring key strengths, when the aforementioned mismatch is better aligned [4].

Globally, autism prevalence is estimated as 1% [5]. Within England, prevalence has been recorded as high as 2.94%, depending on age, with diagnostic rates reducing as age increases [6]. Within medical education, whilst there is increasing recognition of the existence of autistic students and doctors, no published prevalence data currently exist. Within higher education more broadly, however, the number of autistic students is on the rise [7].

Educational outcomes for autistic students in higher education are broadly similar to non-autistic peers [8]. However, difficulties with transitions and poor mental health are common [9]. Within higher education in general, autistic students experience inconsistent reasonable adjustments, requiring repeated disclosures and self-advocacy for implementation, which can lead to feelings of frustration and helplessness [10]. Outside of the classroom, autistic students report challenges with the social aspects of university life, making informal peer support essential in creating safe spaces where they can feel a sense of belonging within their autistic identities [11,12]. To improve the often-negative experiences of autistic students, emerging research recommends increased coordination across university services facilitated by specialist staff, training for faculty on autism, and increased peer support networks – all employing a student-centred approach [12,13].

Within the existing literature, there are few published studies exploring autistic medical students’ and doctors’ experiences. Previous research has demonstrated that autistic characteristics may bring key strengths to the study and practice of medicine [14,15]. Such potential strengths include pattern recognition, problem solving and empathy [16]. Beyond consideration of specific strengths like these, society benefits more broadly from the diversification of the medical workforce [17]. The infusion of such diversity facilitates improved access experiences for those most in need of healthcare services [17]. This is key to equitable healthcare for minority groups. In the case of the autistic community, they experience poorer healthcare outcomes, have a reduced life expectancy, and experience more co-occurring conditions than non-autistic people [18,19]. Whilst no biological cause of such disparities has been identified, the literature is rife with evidence of healthcare barriers as a potential cause. Furthermore, such barriers lead to late presentations of serious conditions and have been statistically associated with adverse outcomes [20]. There is additional growing evidence that autistic doctors and medical students may be better able to communicate with, relate to, and understand the needs of autistic patients [14,21,22]. Whilst these studies are qualitative, and based on the clinicians’ self-perceptions, as opposed to the patients’ perspectives, many autistic doctors will also have a plethora of experience of also being patients [18,19]. The potential benefits introduced by autistic clinicians may help improve the health inequity for this vulnerable population [14,21,22].

The importance of recruiting and, more importantly, retaining autistic people within the medical workforce is therefore paramount. However, evidence suggests that the experiences of autistic medical students and doctors are often far from positive [14,15]. In previous qualitative work, autistic medical students have reported a wide variety of barriers to the successful completion of a medical degree. These spanned from sensory and environmental challenges through to more pervasive systemic issues driven by stereotypical understanding of autism from peers and medical educators [15]. Disclosure of their autistic status to their medical schools led not to support, but rather to negative repercussions and, in one case, attempted expulsion [15]. This was grounded in the concept of weaponised professionalism, whereby stereotypes around autistic empathy and communication skills were seen to be at odds with becoming a doctor, leading to questions of their fitness to practise medicine [15,23,24]. Those who did receive adjustments to their studies found them generic, unhelpful, and unsuited to their personal needs [15]. It is therefore unsurprising that, in a study of 225 autistic doctors, 72% chose not to disclose their autistic status when they were at medical school [14]. Looking at those who did disclose, only half received any adjustments as a result [14]. Despite the many challenges described above, 82% of autistic doctors were currently working successfully as doctors and 74% enjoyed doing so [14]. One key issue, however, seemed to be a sense of isolation. Having never worked with another doctor they thought might be autistic was associated with considering suicide [14]. This supports the importance of role modelling within the medical profession, to help combat any lingering stereotypes that might otherwise negatively impact wellbeing [25].

There is a clear disconnect here between the drive for a diversified workforce, the potential strengths autistic people bring to the medical profession, and their subsequent experiences of medical education. A previous study touched on the benefits of online support, describing this as “life changing”, but did not focus on this in depth [15]. The online support in that case came from ‘Autistic Medical Students’ (AMS), as a sub-group of Autistic Doctors International (ADI). AMS was founded in 2019 as an online support community and includes over 200 members. This community aims to provide members with a safe space for peer support and socialising. Given the negative experiences frequently reported in medical education, and the previous acknowledgement of benefits to AMS membership, this study aimed to explore autistic medical students’ experiences of online peer support, using the example of the AMS group. Sub-aims were as follows:

1. To explore the experiences that led to participants joining AMS, providing contextualisation for their subsequent experiences within AMS.
2. To explore the culture and social dynamics within AMS, how they developed, and how they affect participants.
3. To examine how being a member of AMS influences autistic medical students’ self-perception as a medical student/future doctor.

This paper serves to help establish and contribute to knowledge on the beneficial socio-cultural structures within the AMS community, so that further studies may explore how to transfer these positives into wider medical school and clinical institutions.

## Materials and methods

### Project conception

The need for this study was originally born from conversations between SCKS and MD. Recognising the dearth of research in this area, SCKS designed the initial proposal for this work. He then refined this with input from MD. SC then joined the team for the dissertation component of her medical degree.

### Methodology

This study adopted a qualitative approach. More specifically, our methodology of choice was interpretive phenomenology – a branch of qualitative research, which seeks to provide an in-depth understanding of participants’ lived experience and how they make sense of their reality within their own world [26]. This methodology embraces the subjectivity and the variability of lived experience [27]. Within interpretive phenomenology, researchers’ own positionalities are acknowledged and used to drive the research. This is argued to be helpful in the search for deeper meaning, which is key to the methodology – providing a fusion between the researcher and participants in this process [27]. In our case, we conducted this research from a social constructionist perspective (see ‘philosophy and positionality’ section below).

### Ethics

This study was approved by Brighton and Sussex Medical School Research Governance and Ethics Committee (reference: ER/BSMS9E11/1). Informed consent was audio-recorded prior to interviews (see “data collection” section below). This process was included within our ethical approvals.

### Participants and recruitment

Gatekeeper permission was obtained to conduct the project from ADI. An advert was then posted in the AMS WhatsApp and Facebook groups. The inclusion and exclusion criteria are outlined in Table 1. Interested participants were emailed a participant information sheet by SC and asked to complete a demographic data form. A consent form followed, for them to review.

**Table 1:** Inclusion and exclusion criteria.

Inclusion Criteria	Exclusion Criterion
• Member of AMS. • Current medical student. • Identifies as autistic (self-identified or formally diagnosed).	Any members of the AMS group who are transitioning into residency or foundation training (postgraduate medical training)

Five people participated. All were enrolled on undergraduate medical courses. Three studied in the UK, one studied in Romania, and one studied in Belgium. Further demographics are outlined in Table 2. Our decision to include those self-identifying as autistic – even though none were subsequently recruited – Is increasingly common within autism research, given the demand for autism assessments far exceeds capacity in many parts of the world, which can lead to delays of many years for those awaiting assessment [28]. Private assessments may expedite this but also foster inequity in those unable to afford and receive formal diagnosis. From a social justice perspective, it therefore felt imperative to facilitate the voices of those without formal diagnosis, should they wish to participate.

**Table 2:** Participant demographics.

Participant number	Age	Gender Identity	Current Year of Study/ Total Length of Course	Age Started Identifying as Autistic	Formal autism diagnosis? If yes, life stage at diagnosis	Length of time of AMS membership
1	23	Female	2/6 years	Age 19	Yes, adult	2 months
2	25	Female	4/6 years	Age 20	Yes, adult	11 months
3	30	Female	3/4.5 years	Age 27/28	Yes, adult	1 year
4	22	Female	4/5 years	Age 11	Yes, teen	10 months
5	25	Female	5/6 years	Age 21	Yes, adult	3 years 4 months

### Data collection

Data collection took the form of 1-to-1 semi-structured interviews via Zoom. An interview topic guide (S1 Appendix) was created in advance of this – drawing on the pre-existing literature and the research team’s own experiences as autistic people working in or studying medicine. This process was led by SCKS with iterative input from MD and SC. Participants were sent a copy of this in advance of the interviews, to promote a sense of predictability and accommodate any differing processing needs. Interviews were then undertaken by SC. Each lasted around an hour. Before each interview, informed consent was audio recorded and ^th^ere was an opportunity to ask any questions. Interviews took place between 19^th^ November 2023 and 19^th^ December 2023.

### Data analysis

Interview audio recordings were transcribed automatically by Zoom. Transcripts were then checked for accuracy and corrected by SC. Following this, the transcripts were analysed individually using the Smith et al framework for interpretive phenomenological analysis [29]. This analysis was undertaken by SC and SCKS. They both immersed themselves in the data by re-reading transcripts and re-listening to the interview recordings. They met to analyse the first transcript together in live time. This was led by SCKS, who is well experienced in this analysis method. During this meeting they made initial exploratory notes which were then developed into experiential statements using the comments features on Microsoft Word. Experiential statements were reviewed for connections and organised into personal experiential themes (PETs). Throughout this process SC’s own experience as a member of AMS was significant to the interpretation. She was mindful to acknowledge how her personal experiences may influence what emphasis was placed on different PETs. Once this process was completed for the first transcript, SC independently repeated this for the remaining transcripts, one at a time. SCKS verified this analysis remotely, cross-comparing back to the original transcripts for each participant. SC and SCKS then met again to explore the connections across cases by reviewing the PETs, to construct group experiential themes (GETs).

### Philosophy and positionality

At its core, this study sits within a social constructionist paradigm [30], combining a relativist view of ontology and a subjectivist view of epistemology. It is, however, important to dig a little deeper than this when undertaking social justice informed research. Were our epistemological considerations to truly end at discussion of socially constructed knowledge, we would neglect a key strength within this work. This study is an example of an all-autistic research team, undertaking insider research to advance understanding around the social world of autistic medical students. This positionality is important and ties in with the concept of epistemic injustice. Epistemic injustice refers to injustices in the way human knowledge is created, which is influenced heavily by power (the powerful are seen as credible) and privilege (the privileged are afforded the opportunity to undertake and publish research in high impact journals) [31]. A lack of insider representation has indeed reinforced a narrow field of view in autism research historically, reinforcing outdated stereotypes and stifling the publication of insider research offering a challenge to such discourse [15]. The positionality of our research team is a prime example of the increasing drive to combat such epistemic injustice. Table 3 outlines our overarching philosophical and methodological framework in this context.

**Table 3:** Philosophical and methodological framework.

Ontology	Relativist
**Epistemology**	Subjective, constructionist, justice focused.
**Paradigm**	Social constructionist
**Approach**	Qualitative
**Methodology**	Interpretive phenomenology
**Data collection methods**	Semi-structured interviews
**Data analysis methods**	Interpretive phenomenological analysis

## Results

Our analysis constructed a variety of GETs, which were temporally divided into pre-AMS and post-AMS discovery. The pre-AMS GETs explored the ableism in medical culture (GET1), the experience of role conflicts between being both autistic and being a medical student (GET2), as well as challenges with isolation (GET3) and weaponised professionalism upon disclosure of their diagnosis (GET4). Whereas the post-AMS themes reflect the participants’ journeys in finding similar people and a sense of belonging (GET5) in a safe, validating space for support (GET6), whilst realising their autistic strengths, such as problem solving (GET7), and developing courage to advocate for accommodations for their unique needs (GET9). Participants recognised that they could thrive in medicine (GET8) and benefited from seeing the success and challenges of their peers, and of ADI doctors, instilling a sense of optimism for their futures (GET10). The post-AMS GETs align with sub-aims 2 (to explore the culture and social dynamics within AMS, how they developed, and how they affect participants) and 3 (to examine how being a member of AMS influences autistic medical students’ self-perception as a medical student/future doctor) as outlined in Table 4.

**Table 4:** Group Experiential Themes. SA = sub-aim.

Pre-AMS GETs	Post-AMS GETs
1 – Medical Culture **(SA1)**	5 – Finding their people/neurokin **(SA2)**
2 – Is it a stereotype or is it true? **(SA1)**	6 – Safety **(SA2)**
3 – Difference is dangerous **(SA1)**	7 – Not destined to fail **(SA2/3)**
4 – Don’t I deserve help? **(SA1)**	8 – Internalised optimism for the future **(SA3)**
	9 – Confidence to make it happen **(SA3)**
	10 – Real modelling **(SA3)**

### GET 1: Medical culture

All participants’ experiences of medical culture reflected a need for perfectionism. This ideology was not limited to clinical competence but included socially interacting in a neurotypical way. Difficulties were not discussed or disclosed amongst peers.

*“There is like a constant pressure on medical students to be strong, be good” (P2).*

The underlying attitudes of how medical students should act, and not require support to work as doctors, fostered perceptions of a wider culture of systemic ableism against all disabled students.

*“I got the comments such as, ‘how are you going to be a doctor, if you need X, Y and Z’” (P3).*

Ableism was central to most reported experiences here, with participants feeling like broken versions of medical students. This was reinforced through poor experiences of support. For example, when participants reached out to their medical schools, some were offered generic adjustments that ticked a box but did not lead to equitable experiences.

*“I sought general support from the university, um wellbeing service um I can’t say it was particularly helpful” (P4).*

When advocating for more specific or personalised support, some reported being treated badly, invalidated, or discriminated against, resulting in one having to seeking external support in challenging their medical school.

*“I actually had to get the BMA (British Medical Association) involved at one point to help me out because I was having such a poor experience with support” (P3).*

When there were deviations from expectations, participants were left to suggest what would support them with their studies. Participants experienced resistance to implementing specific and useful learning adjustments from medical schools.

*“There are some people who will just, um, kind of shut it down immediately, without actually considering, like, what the issue is” (P1).*

### GET 2: Is it a stereotype or is it true?

Participants’ medical schools lacked knowledge about the heterogenous complexity of autistic traits.

*“I was hearing lots of people, um, talking about autism in a sort of a generally negative way” (P1).*

Academic literature used to complete assignments at medical school conveyed perspectives to ‘cure’ or ‘treat’ autism.

*“…you don’t have to cure us… it’s very, very frustrating…it’s all very ableist. And it’s insulting to me” (P5).*

Long-standing inaccuracies that autistic people lack empathy were well known by participants. This stereotype had influenced non-autistic peers and one participant experienced direct discrimination about her behaviour, because of this myth.

*“[A non-autistic peer] was saying, ‘well, now, we know who the autistic one is’” (P3).*

The negative messages being received by participants led to them question their ability to become doctors. Imposter syndrome and helplessness were commonplace.

*“I couldn’t- cause I didn’t really know what autism was until I got diagnosed. And um I felt like, “Can I even do this kind of job when I’m autistic?” (P5).*

Furthermore, participants experienced role conflicts where being an autistic person often meant they had poor experiences with healthcare professionals, but being a medical student meant they were working in a profession notoriously ableist towards autistic people.

*“But I think there is always that little bit in your brain that doesn’t outright say (you’re autistic) because you don’t- because medical staff are… also people with a lot of stigma” (P3).*

### GET 3: Difference is dangerous

Participants experienced feeling different.

*“My classmates talk about … their issues… they’re not necessarily my issues” (P2).*

They struggled to fit in with their peers and find meaningful connections, despite attempting to mask or camouflage their autistic identities.

*“It was because I felt lonely, I think. And I felt like I was like an ugly duckling, the odd one out like, you know?” (P5)*

This led to isolation and burnout. Social isolation had negative impacts for mental wellbeing as well as academic attendance.

*“…not having made strong social connections… was definitely a barrier... I think if I’d been like less embarrassed about it, and had like someone to sit with, it, would have made it much easier to (attend)” (P4)*

Disclosing autism to peers and the university was a considered decision, carefully weighing up the pros and cons, which was an energy-consuming process.

*“There’s a lot of stigma... I feel like there’s a lot more to lose in terms of how lecturers and peers see you”* (P2)

Participants did not disclose their autism diagnosis for fear of consequences from peers and staff. These negative associations promoted a sense of shame. A culture of silence about being autistic was embedded in their experiences.

*“It’s almost like… if you have it, then then it’s a dirty secret, and you don’t let people know” (P1).*

### GET 4: Don’t I deserve help?

Most participants only disclosed their diagnosis to universities when needing support. This, however, reinforced stereotypes.

*“If you only disclose or mentioned your autism when you find something difficult – some people get this kind of association between autism and it stopping you from doing things” (P1).*

Reasonable Adjustments (RAs) to their medical studies were often denied by medical schools. For some participants, this felt like an uphill battle.

*“I feel like I’m constantly putting in all this effort to advocate for myself to get like… accommodation and basic human rights” (P2).*

If there was a deterioration in a participant’s mental health, meetings were held, where ableism and weaponised professionalism were rife.

*“People’s immediate response is ‘oh, no, that’s not professional. You can’t wear ear plugs. People will think that you’re not listening to them’” (P1).*

*“My university has a poor track record of conflating wellbeing concerns with professional concerns” (P3).*

These interactions led to internalised ableism and shame, in a time where participants were already in need of support.

*“Getting comments from occupational health of ‘Why do you need that? How are you going to be a doctor?’… it fuels that imposter syndrome, and you just invalidate yourself” (P3).*

Asking for support and advocating for help was fundamentally difficult for some participants due to the trauma of growing up undiagnosed.

*“I think, growing up autistic, but not knowing um often, you want to kind of make yourself small and not draw attention to yourself.” (P1)*

The experiences with lack of RAs, being invalidated and dismissed as inadequate when asking for support, led to helplessness about the future.

*“I think I suffered a lot from that feeling of invalidation from the support, because at the time that I thought, this is only people I have. And they’re invalidating me and not supporting me or providing the support I need. Where else am I supposed to go?” (P3)*

Subsequently, participants continued to mask their autistic traits on placement.

*“What does this person want me to say right now? And what is like socially acceptable? And how am I supposed to react to what they are saying? And I have to smile now, and now I have to, you know...it’s like very calculated communication” (P5).*

The consequences of the lack of RAs, ableist statements and continued masking led to burnout.

*“By summer, I was so burnt out that… for the first time, I was dealing with like suicidal thoughts” (P3).*

### GET 5: Fin ding their people/neurokin

The aforementioned experiences led participants to seek support through joining AMS. Upon doing so, participants were initially unsure about the social rules of the group. They observed conversations before actively participating themselves.

*“So it’s a bit like confusing at first… with like the social cues” (P2)*

During this initial observation period, participants recognised themselves in other members with respect to direct communication styles and attention to detail.

*“Part of the validation was seeing a lot of people type and talk in the way that I do like, you’ll see a lot of long messages, and I like to give lots and lots of context and lots of detail and it was nice to be in a group chat where other people were doing that as well and really engaging with each other” (P3).*

For the first time, participants were finding other people with similar experiences, and behaviour to them, in a transparent and open environment.

*“Even to say… something… that you wouldn’t say in real life and say, “oh, I had a meltdown today”… I could never say that, but I* ***can*** *say that* ***here****, and like that’s great stuff, and it makes it less scary to acknowledge it, at least” (P2).*

They felt a positive sense of truly belonging and had found their people.

*“I feel like it’s a lot more safe, cause I feel like I’m surrounded by people like me” (P3).*

*“It gave me a lot of courage… I felt a sense of… belonging somewhere” (P5).*

### GET 6: Safety

Participants traditionally anticipated hostility and being misunderstood when interacting with neurotypicals.

*“Neurotypical people will perceive as um irritated or kind of um passive aggressive” (P1).*

However, within AMS participants related to the struggles that other members recalled.

*“It became quite apparent that actually a lot of people struggled, or they were having all the same thoughts about- like there was different anxieties and worries - that I was having” (P4).*

In vulnerable times, where participants had been invalidated by colleagues or institutions, or were confused by the responses of others to their actions, they shared these experiences with the group.

*“I also sent something about um my professor being very ableist…It was like kind of shitty to hear that other people at the same experience. But then, like also like very affirming of, you know, I’m not crazy” (P5).*

Other AMS members validated what participants had experienced, had empathy and understanding and could offer practical advice on how to manage difficult situations when appropriate.

*“I think imposter syndrome is always part of being medical student and a doctor, but as far as an autistic medical student, again, the validation is really helpful, and it’s made me feel a lot better about my place in medical school, and. you know, working through the difficulties I’ve had” (P3).*

This led to participants agreeing that AMS was a safe space to share invalidating experiences and difficulties.

*“I know now that I can contact anyone and ask for some help or advice, and I don’t need to rely on my school, because I think at the time I didn’t think where else I could go or who else can I speak to without being invalidated” (P3).*

Additionally, many felt that just knowing that AMS exists and was an accepting group that they could go to in times of needs added an element of comfort. It meant they were not alone in fighting for support and acted as a safety-net in their day-to-day lives.

*“Even though I don’t speak to many people a lot, just the group existing and being with so many members. It’s just gives a sense of, you know I’m valid, and I’m supposed to be where I am” (P5).*

### GET 7: Not destined to fail

The AMS support group was reported to be different from other autistic or medical student support groups. The intersection of these identities makes AMS unique.

*“You actually want advice from someone that gets your perspective that without like putting on all the disclaimers” (P2).*

*“With somebody who is a medical student, and is autistic, the advice that they give so often is much, much more relevant to me” (P1).*

The discussions participants experienced when asking for advice were solution focused.

*“You’re all working together to improve your situation rather than kind of dwelling on um that you’re in a really negative position” (P1).*

The validation and problem-solving approach to participant’s concerns gave them plans and hope for the future – for change in managing the situation should it recur, and for systemic change in attitudes.

*“It generally tends to be a very… optimistic kind of discussion” (P1).*

These experiences combat the tragedy narrative which often occurs for participants and makes achieving their degree and becoming an openly autistic doctor seem possible.

*“People were talking about it (difficulties) openly, and that was quite refreshing” (P4).*

*“I feel like the way to move things forwards is for people, when they have interactions with autistic people, to know that those people are autistic, because otherwise they get a very kind of one-sided view of autism” (P1).*

### GET 8: Internalised optimism for the future

Due to the wide range of academic year groups, as well as up to two-year-postgraduate doctors, in AMS, participants saw a future in medicine.

*“I think sometimes it’s difficult not to feel like this is too much of a challenge. Am I ever gonna be able to cope with it? And will I manage to work as a doctor? Um being able to have those things that… make it feel easier, can kind of make you feel more optimistic for the future” (P1).*

Participants felt they could survive within medical culture.

*“It shows that there are lots of people out there and like people find different ways of coping. So even when you’re struggling, you know that like… it is possible? And there are other people out there that you can talk to if you are having any difficulties” (P4).*

Not only could participants survive, but also thrive in medicine.

*“It’s really nice to see people that are thriving in medicine and like it makes me more confident that I really do want to do this, and that I can do it” (P2)*

Many members of AMS had varying routes into medicine and had taken time out of training. This helped one participant broaden her horizons on what medicine and her life could look like for the future.

*“The biggest impact for me was, um, seeing a lot of, erm, people that went into medicine later, like autistic people that were like, ‘oh, I dabbled here and there and stuff, and now I’m doing medicine’ or ‘I took time off and like I came back to it’” (P2).*

Participants had previously worried that speciality choices would be restricted due to their autism, but now feel able and confident that they could excel in any chosen speciality.

*“Just seeing people like being anaesthesiologist, or a psychiatrist or a general physician. Just, you know, all the specialties…It just made me see that I could do what I wanted to do” (P5).*

They recognised their autistic strengths and how these traits added benefit to the doctor-patient interaction, such as hyper-empathy, detailed medical knowledge and observant natures.

*“Often autistic people are very good at coming up with creative solutions to their problems” (P1)*

*“I think of myself as a very empathetic person like, I love talking to people, and I love, you know, talking about their feelings…And then I saw a lot of people talking about the fact that in one-on-one with the patient they have like very same thing like they’re very good at communication and very empathetic” (P5).*

One participant recognised how autistic strengths can flourish in well-supported environments.

*“If you’re well supported, you can work properly, and you can use your strengths that you have because of your autism” (P1).*

### GET 9: Confidence to make it happen

From the discussions observed, and conversations in the group, participants started to recognise their own needs – and how medical school and hospital environments were impacting upon these.

*“Having a better understanding of what other people find difficult can help me work out what I might be finding difficult” (P1)*

Participants then accepted that they had different needs to non-autistic people and that these needed to be met.

*“I learned to accept that I am doing the best I can with my abilities, and I don’t have to prove myself to anyone, and I don’t have to compare myself to anyone because they are not living my life, and they are not having my struggles, and vice versa” (P5).*

Some participants asked their medical schools for support to meet their autism-specific needs. This was often refused, even with further self-advocacy, so they self-implement adjustments.

*“I got more creative in the terms of what I can do myself. It, instead of like going to them and saying, “Hey, can I have this in place and stuff like that?” because I don’t think they’re involved with the student body in that particular order” (P4).*

This included self-advocacy on placements.

*“So now that every moment I find the place to sit down, I just sit down, I don’t even ask for permission. I’m just like… I’m just gonna sit down unless someone tells me to move” (P2).*

One participant went beyond accommodating her own needs and, via an opportunity advertised on the AMS Facebook page, did a disability rights course. This enabled her to advocate for autistic students in medical education on a wider scale.

*“I actually found out about Disabled Students UK’s consultants training, I think, from the group being posted on Facebook, I think there’s an advertisement for it… It’s nice to see that there’s actually a place for this. And you know, there’s something to look forward to with my career” (P3).*

### GET 10: Real modelling

Participants benefited from seeing the successes and challenges of their peers, and of newly qualified doctors.

*“There are students like that are… really struggling, but like then I’ve seen them overcome the struggle, and that, like also is like super inspiring and helpful cause it makes you feel like a bit better” (P2).*

Participants also benefited from role models in ADI, as doctors further into their careers – providing aspirations in the longer term.

*“It’s really nice to see people that are thriving in medicine and like it makes me more confident that I really do want to do this, and that I can do it” (P2).*

Prior research produced by the ADI team had communicated in written form about the assets of autistic doctors and had given participants validation that they belong in medicine.

*“I’m quite interested in the idea of peer support for um doctors as well. So, I do like the academic side of it um and keeping up with- I know the medical student group is kind of an offshoot of… the doctors group- but the main doctors group do quite a lot of research... And so that’s been really helpful to me in terms of my kind of like my learning of autism, and and the ways that that we can change our system to be more inclusive” (P1).*

*“When I hear other autistic people in the-. especially in the medical field, like people researching it and living it and they say the exact same things that I’m saying…” (P5)*

Reported experiences also stepped beyond traditional role modelling into sense of ‘real’ modelling. The reality of difficulties and barriers autistic doctors faced, alongside the impacts on mental wellbeing, were not downplayed or trivialised for participants.

*“It helps to have people that that you see, who are doing it, um almost like role models… [but] people share their difficulties, because sometimes… you’ll see a role model, and it seems almost unattainable, because they’re only showing you the positive aspects of it… and you know with yourself that you have all of these challenges, whereas when you’ve got somebody who’s doing it, er, but actually admitting to the things that are difficult and working through things… Then it kind of, um, shows you that it is possible… to work through these challenges” (P1).*

Participants were inspired to be open about their diagnosis and traits when not in crisis – to real model to others, and reduce the stigma associated with autism, shifting the focus to autistic strengths.

*“I’ve always felt that if you behave …as though you’re ashamed of something, then you then you send the message that it’s something to be ashamed of… and for other autistic people… it was important for me to… not act as though I’m ashamed of it” (P1).*

*“Everyone thinks of me as a weird, odd thing. Umm but just seeing other people, like owning it, made me want to own it” (P5).*

One participant stepped beyond this and wanted to improve the wider healthcare system for autistic patients.

*“It’s made me more… passionate to kind of like, seek out… ways of changing the (healthcare) system to be more supportive of, um, autistic people” (P1).*

## Discussion

The results of this study reflected those of previous studies analysing autistic medical student and doctors’ experiences [14,15]. Participants experienced systemic ableism – practices that exclude disabled people from full participation and equal opportunity, in different ways. For example, participants’ requests for support were dismissed as being incompatible with becoming a doctor when they sought RAs for their autistic differences. The lack of understanding from medical schools led to hopelessness and, with nowhere to go for support, participants internalised the views of their institutions – that they were inherently flawed due to their disability. This is a form of internalised ableism. Fortunately, this study revealed new positive experiences with respect to the AMS support group. Participants felt as though they belonged and were no longer alone in their struggles or ways of communicating. Many participants benefited from the solution-focused discussions and applied this learning to their own medical studies. They became more accepting of their own needs and implemented self-accommodations. ‘Real modelling’ – where both the challenges and successes of a role model are displayed – allowed participants to have hope for the future and feel able to thrive in medicine.

The harm caused by the lack of implementation of specific RAs may be considered a type of epistemic injustice. Epistemic injustice is where a person’s knowledge or ability to describe their experiences is discredited (testimonial injustice), or when someone’s experience is poorly understood as they do not fit concepts known to themselves or others (hermeneutical injustice) [32]. An example of hermeneutical injustice is that medical schools may be unaware of the best RAs to support autistic students, with little information available about their experiences, so cannot implement them. This is reflected in studies exploring higher education in general where staff reported high levels of anxiety about implementing RAs for disabled students [33]. On the other hand, participants’ disabilities may have been discounted, where participants’ needs were not considered to be autistic traits that required accommodations but instead trivialised to the individual’s own personality or an inherent weakness – a type of testimonial injustice [34].

These injustices led to participants advocating for individual support. Many also participated in diversity work to improve awareness and acceptance of autism in medical education. This takes time and resources away from studying, career development and socialising, which may disadvantage students. This deviation of resource due to disability is called minority tax [35]. Despite this additional burden, studies on minority tax in medical schools have found that the learning environment can reduce its effects [36]. For example, peer autism training may prevent bullying and further stereotyping, and updating curricula and resources could reduce the need for regular advocacy from students when lectures contain outdated knowledge [36]. Medical schools accessing disability training may help them to understand and work synergistically with students to form appropriate RAs [37].

**Fig 1:**
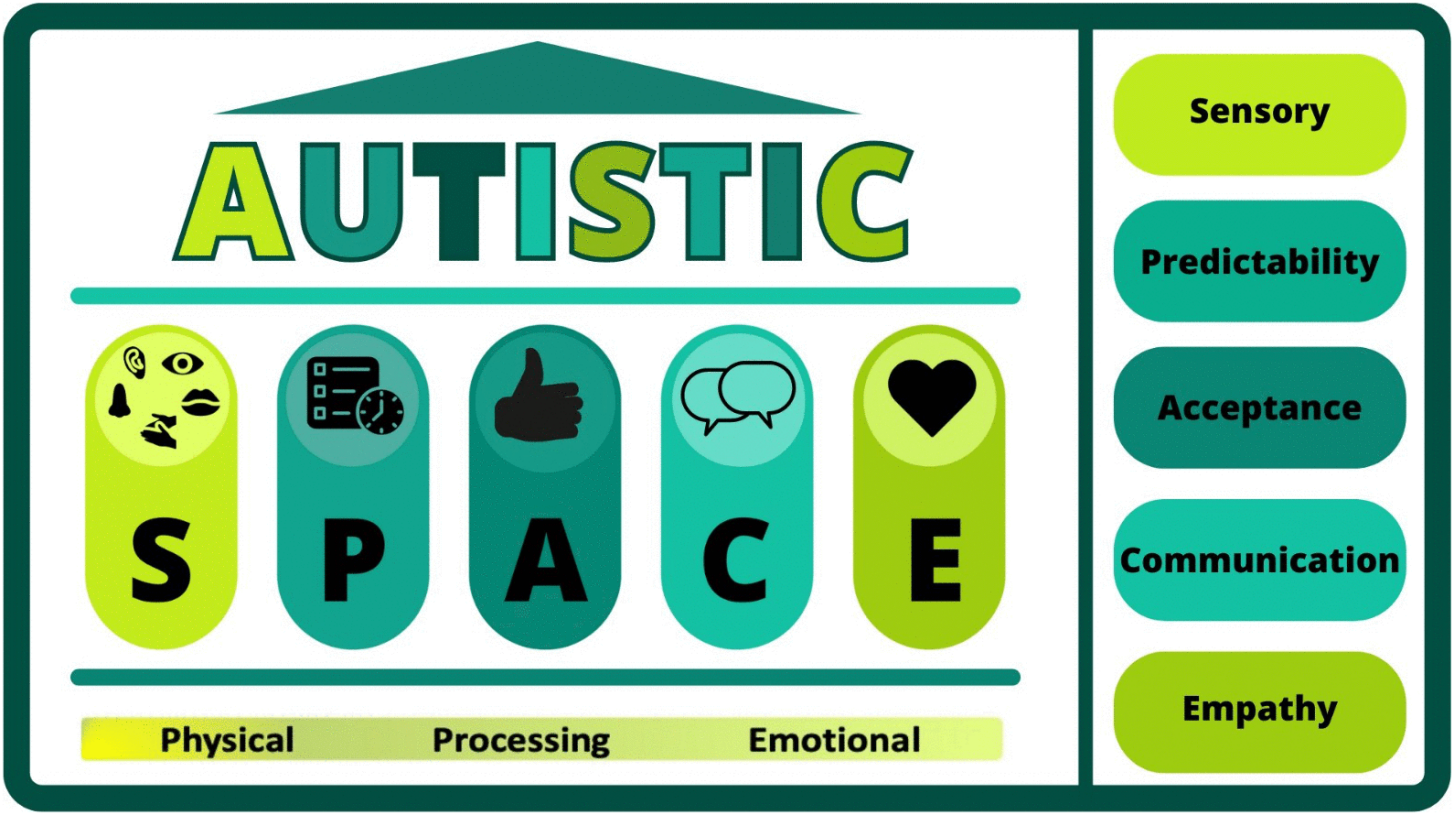
Autistic SPACE framework [38].

Many participants discussed how beneficial solution-focused discussions were in AMS. These often related to how to adapt to sensory challenges or manage communication differences. The Autistic SPACE framework, created to support the needs of autistic patients in healthcare settings, could be of transferable use for autistic medical students and their institutions [38]. Fig 1 outlines the elements of the Autistic SPACE framework [38]. Many discussions in AMS centre around these five needs already, but if medical schools adopted this approach when discussing RAs collaboratively useful support may be implemented. This is supported by Dexter et al who concluded that more individualised support from staff-student collaborative work would be beneficial and remove the burden from students to conceptualise and implement their own adjustments [39]. Furthermore, this approach would align with the UK General Medical Council’s guidance that disabled students should be consulted to find the most appropriate RAs for their individual circumstances [17].

Pre-AMS, participants experienced difficulties forming friendships from social interactions with neurotypical peers. Previously, these differences had been attributed to a deficit in autistic people’s social communication skills [40]. More recently, Milton suggested a different view of these interactions called the ‘double empathy problem’ [41]. The double empathy problem dictates that mismatches in communication between differing neurotypes are the responsibility of both autistic and non-autistic people, as both struggle to understand, empathise and relate to each other [41]. This can lead to cross-neurotype miscommunications. Once our participants joined AMS, they did indeed relate more easily to other members’ communication styles and formed social connections. This is reflected in wider research that has found that not only is autistic peer-to-peer communication effective, but that being around other autistic people can be a highly positive, life-enhancing experience for autistic people [42,43]. Specifically, university students have reported ‘autism societies’ enable a physical space to meet peers they can relate to and develop friendships [44].

This relatability gave our participants a sense of belonging. This is instrumental in developing social identity – a person’s sense of who they are based on the groups they identity with – which interplays with a person’s self-concept, how someone perceives or evaluates themselves [45,46]. A systematic review concurred that people developed a more positive autistic identity when receiving external autism acceptance and support, which in itself was associated with improved mental health [47]. A different study assessing social identities in autistic people found that connection to other autistic people was associated with better mental health outcomes [48]. Therefore, members of AMS may have improved wellbeing and increased confidence in their self-identity, because of the autistic peer connections in the group.

Participants found masking with neurotypical peers exhausting, which contributed to autistic burnout. The risk of burnout can be reduced by unmasking and accepting autistic needs [49]. Unmasking is a process of self-discovery about autistic needs, and stopping masking behaviours to embrace their authentic, autistic self [45]. However, this knowledge acquisition can be burdensome, with a previous study participant reporting how the introspective knowledge discovery about autism was like an ‘additional degree’ [15]. The AMS group may facilitate some of this knowledge exchange as a space to ‘unmask’, in turn reducing this learning load. There are almost no publications related to the impact of unmasking on autistic people, likely primarily due to the barriers of doing so. Anecdotally, it is often unsafe for autistic people to unmask; this risk can vary depending on other intersections of their identity such as gender or race [45]. However, Botha suggests that autistic community connectedness helps to combat internalised ableism and reframe how someone views themself [50]. AMS may provide an important space for autistic medical students to share knowledge, unmask and develop acceptance and autistic pride.

A distinct finding from our study was that AMS provided a unique space for people who were both medical students *and* autistic. Autistic people often mistrust medical professionals due to experiences of feeling misunderstood, therefore being a medical professional may be perceived as at odds with being an openly autistic person [21]. Whereas studies show general medical students feel unprepared in adapting clinical practice for autistic patients, with doctors often misunderstanding autistic peoples’ needs [21,51]. This creates role conflict for autistic medical students, where conflicting demands are placed on them due to their different societal identities [52]. Within AMS, however, our participants were able to appreciate both sides of these experiences and support each other through this discomfort.

Whilst participants associated strengths with being autistic, most had experienced ‘learned helplessness’. Learned helplessness is when there is a perceived lack of control about the future, usually occurring after repeated exposure to stressful situations where needs have been dismissed, or efforts unrewarded [53]. For our participants, their needs were repeatedly dismissed through experiences of weaponised professionalism. Fortunately, AMS marked the discovery of openly autistic role models. Not having role models as a minority group has been shown to be disadvantageous. Role models are needed to motivate and inspire students, to help them develop a professional identity, and to remove barriers to success [54]. Within AMS itself, experiences are grounded in realism, where inspiration from peers or doctors is seen through ‘real modelling.’ This ensures that autistic medical students can appreciate the challenges their seniors have faced, whilst celebrating their successes, and relating to the experience [16]. The need for openly autistic doctors, across demographical groups, is evident and creating a wider medical culture where this is possible from earlier stages within training would be beneficial for both patients and colleagues [14,15].

### Strengths and limitations

Within interpretive phenomenology, there is an underlying assumption that the researchers are experts within their study area, which allows them to be specific in how best to obtain useful knowledge from participants [27]. This strength was seen throughout this study, with pre-interview study guides, acceptance of stimming and breaks in interviews, and email reminders to help with differences in executive function. Whilst the study only recruited five participants, this enabled an in-depth exploration and analysis and meant that we could extract how they made sense of their own experiences through both pragmatic and emotional lenses.

Our sample of participants is also key to consider here. All identified as female and had formal diagnoses of autism, all bar one of whom received their diagnosis in adulthood. Traditionally autism research was dominated by male participants, likely stemming from a lack of recognition of autism in those who were not cisgender males [55]. In recent years, however, this discrepancy has been increasingly narrowing [55]. Similarly, as autism awareness grows, it is increasingly common for people to receive an autism diagnosis in adulthood [56,57] – and in those who are not cisgender male, this may be associated with de-diagnosis of a personality disorder or other psychiatric labels, all of which may delay the journey to receipt of subsequent autism diagnosis [55]. Whilst there is no study of the broader AMS membership to formally explore such demographic factors, there has been such a study for the ADI (doctors) group in 2023. That study found that 81% of respondents identified as female, 64% had a formal diagnosis, and of those with a formal diagnosis, 94% received this in adulthood [14]. This would suggest, as much as we can ascertain, that our sample in this present study seems reasonably reflective.

Our own positionality is also an important reflection here. All three of us are members of ADI, with MD and SCKS being in key leadership positions within the group. SC is a member of AMS. It is also worth noting that none of us know any participants ahead of the interviews. To reduce any ‘upward distortion’, where individuals in a lower-status position (medical student) speak of experiences more positively to those in a higher-status position (consultant) than they would to peers, participants were interviewed by SC, who is a fellow medical student. Whilst this shared identity may have facilitated an open and honest dialogue, over-identification with participants experiences could lead to poor critical analysis of data. This is where SCKS’s role in the analysis provided an important alternate perspective. The dual role of MD as ADI founder and co-author raises the possibility for confirmation bias, where positive outcomes are unintentionally emphasised. To mitigate this, MD did not directly engage with participants or with the analysis.

The study is qualitative. Therefore, the findings are not generalisable but may be transferable depending on the readers’ experiences. It may be that participants engaged with the study due to having particularly extreme negative experiences (a volunteer bias) and therefore do not represent a wider experience. However, the experiences between participants had congruent themes. This study does not include AMS members who left medical school or those who left AMS, which future studies could explore. Additionally, all participants were female, so we cannot be certain of the influence their gender had on their experiences. Future studies could explore experiences across the range of genders, alongside wider elements of intersectionality such as race, physical disability, and socio-economic status.

## Conclusions

Participants reported positive experiences being a member of AMS. They felt a sense of belonging, had solution-focused discussions for overcoming challenges, and optimism for the future. However, despite these positive experiences within the support group they also reported discrimination, a lack of understanding and awareness about autism and inappropriate RAs from their medical schools. Participants benefited from real modelling between peers, and autistic doctors, and identified strengths with being autistic. The open and accepting culture of AMS to discuss difficulties – often a rarity within medicine – had enabled participants to develop their sense of social identity, accommodate their needs and thrive as autistic medical students. Further exploration into how to transfer these phenomena from AMS to wider medical culture is needed, to continue to improve autistic medical students’ quality of life and enable them to become assets to medicine.

## Supporting information

S1 Appendix Interview topic guide. (DOCX)
