# Supplementary material for: The experiences of autistic medical students in relation to seeking and receiving online support: A phenomenological study
Source: PLoS One. 2026 Mar 20;21(3):e0345156. doi: 10.1371/journal.pone.0345156 (PMC13004384; doi:10.1371/journal.pone.0345156)
Supplement: S1 Appendix — (DOCX) [file pone.0345156.s001.docx]

**Interview topic guide**

Section 1: Background medical school experiences

- Before you joined the “Autistic Med Students (AMS)” group, did your medical school know that you were autistic? Can you please explain the reasons for your answer?
- Before you joined the “Autistic Med Students (AMS)” group, what were your experiences in relation to adjustments or supports from your medical school?
- Before you joined the “Autistic Med Students (AMS)” group, were your medical studies impacting your wellbeing or mental health in any way? Can you please explain?

Section 2: “Autistic Med Students (AMS)” group experiences

- What led to you joining the “Autistic Med Students (AMS)” group?
- How did you feel when you discovered that the “Autistic Med Students (AMS)” group existed?
- Can you tell us about your early experience of joining the “Autistic Med Students (AMS)” group?
- Which platforms do you use to interact with other members of the “Autistic Med Students (AMS)” group?
- Thinking about your experiences of interacting with other members of the “Autistic Med Students (AMS)” group…
  - Firstly, what are your experiences of communication styles in the group?
  - Secondly, what are your experiences around the sort of topics that are discussed in the group?
- Would you like to discuss any other experiences of the “Autistic Med Students (AMS)” group itself that we have not already asked about? If so, please explain.

Section 3: The impact of “Autistic Medical Student (AMS)” membership

- How has being part of the “Autistic Med Students (AMS)” group impacted your wider medical studies?
- Has being part of the “Autistic Med Students (AMS)” group impacted your disclosure choices? Can you please explain?
- Has being part of the “Autistic Med Students (AMS)” group impacted your experiences around supports or adjustments to your medical studies? Can you please explain?
- Has being part of the “Autistic Med Students (AMS)” group influenced your wellbeing or mental health? Can you please explain?
- Has being part of the “Autistic Med Students (AMS)” group influenced your thoughts or beliefs about autistic people studying medicine or working as doctors?
- Has being part of the “Autistic Med Students (AMS)” group influenced your own confidence or sense of belonging within the medical profession?
- Have you gained any particular insights (or experienced any "lightbulb" moments) by observing other students discuss difficulties they are experiencing in the group?
- What is the most useful practical tip you have learned through being a member of the “Autistic Med Students (AMS)” group?
- Has being part of the “Autistic Med Students (AMS)” group influenced you in any other ways that we have not already asked?
